# Short-term exposure to JUUL electronic cigarettes can worsen ischemic stroke outcome

**DOI:** 10.1186/s12987-022-00371-7

**Published:** 2022-09-09

**Authors:** Ali Ehsan Sifat, Sabrina Rahman Archie, Saeideh Nozohouri, Heidi Villalba, Yong Zhang, Sejal Sharma, Yashwardhan Ghanwatkar, Bhuvaneshwar Vaidya, David Mara, Luca Cucullo, Thomas J. Abbruscato

**Affiliations:** 1 Department of Pharmaceutical Sciences, School of Pharmacy, Texas Tech University Health Sciences Center, 1300 S Coulter St, Amarillo, TX 79106 USA; 2 Oakland University William Beaumont School of Medicine, O’ Dowd Hall, 586 Pioneer Dr, Room 415, Rochester, MI 48309 USA

**Keywords:** Cerebrovascular, Blood–brain barrier, Vaping, Oxidative, Inflammation, Endothelium, Alternative, Exposure, Smoking

## Abstract

**Background:**

The short and long-term health effects of JUUL electronic cigarette (e-Cig) are largely unknown and warrant extensive research. We hypothesized that JUUL exposure could cause cerebrovascular toxicities impacting the progression and outcome of ischemic stroke comparable to tobacco smoke (TS) exposure.

**Methods:**

We exposed male C57 mice to TS/JUUL vapor for 14 days. LCMS/MS was used to measure brain and plasma nicotine and cotinine level. Transient middle cerebral artery occlusion (tMCAO) followed by reperfusion was used to mimic ischemic stroke. Plasma levels of IL-6 and thrombomodulin were assessed by enzyme-linked immunosorbent assay. At the same time, western blotting was used to study blood–brain barrier (BBB) tight junction (TJ) proteins expression and key inflammatory and oxidative stress markers.

**Results:**

tMCAO upregulated IL-6 and decreased plasma thrombomodulin levels. Post-ischemic brain injury following tMCAO was significantly worsened by JUUL/TS pre-exposure. TJ proteins expression was also downregulated by JUUL/TS pre-exposure after tMCAO. Like TS, exposure to JUUL downregulated the expression of the antioxidant Nrf2. ICAM-1 was upregulated in mice subjected to tMCAO following pre-exposure to TS or JUUL, with a greater effect of TS than JUUL.

**Conclusions:**

These results suggest that JUUL exposure could negatively impact the cerebrovascular system, although to a lesser extent than TS exposure.

**Supplementary Information:**

The online version contains supplementary material available at 10.1186/s12987-022-00371-7.

## Introduction

Tobacco smoking (TS) causes more than 480,000 deaths in the United States (US) by contributing to many diseases, including cancer, lung diseases, cardiovascular and cerebrovascular disorders [1]. Although there has been a steady decline in cigarette smoking among adults in the US from 42 to 14% between 1964 and 2019 [1, 2], the increasing use of alternative tobacco products like electronic cigarettes (e-Cigs) poses a new threat to public health. E-Cigs were first introduced in the US market in 2007, and their popularity has risen since [3]. Vaping, the common term for smoking e-Cigs, has increased significantly in adult and adolescent populations [4, 5]. Nicotine is delivered in aerosol form by e-Cig devices produced from a vaporizing liquid. JUUL is a recently developed portable e-Cig device that physically resembles a universal serial bus (USB) flash drive, a feature unique from other e-Cig products in the market [6, 7], which currently is one of the most popular e-Cig brands in the US. Although JUUL e-Cig was introduced to help adult heavy smokers quit smoking or as a less harmful alternative to TS, it is also very popular in adolescents. JUUL e-Cig consists of a liquid & heating coil-containing pod and a rechargeable battery. The nicotine in the JUUL-pod is claimed to be salt-based instead of the free base found in other e-Cig products [7, 8], which could facilitate the vapor inhalation process and generate higher nicotine concentrations [9]. This could make JUUL more harmful to its users than other e-Cigs. Rigorous research is required to elucidate the health effects of JUUL e-Cigs.

Stroke is another major cause of morbidity and mortality in the US, causing death every 4 min [10]. Stroke is primarily of 2 types: ischemic and hemorrhagic. Ischemic stroke comprises 87% of all strokes and is characterized by the interruption of blood flow to the brain [10]. Smoking is one of the most common comorbid conditions that can increase the risk and worsen the outcome of an ischemic stroke event [11]. Our lab has previously shown that exposure to nicotine and smoking can worsen brain injury and neurological outcomes [12, 13] and decrease brain glucose transport [14] and utilization [15] in ischemic stroke. The blood–brain barrier (BBB) is an integral part of the brain neurovascular unit and plays a vital role in maintaining normal brain physiology and ionic and nutrient balance. BBB disruption, inflammation, and oxidative stress are major pathological hallmarks of ischemic stroke [16]. The deleterious role of TS on BBB function, inflammation, and oxidative stress has been depicted in preclinical studies [12, 17–19]. Exposure to nicotine-containing JUUL e-Cigs is predicted to adversely affect the ischemic brain, leading to a poor clinical prognosis.

Some studies have investigated the cerebrovascular effects of e-Cigs [12, 15, 20], but very few [21] have specifically addressed the toxic effects of JUUL e-Cigs on the brain. Ramirez et al. have shown that short-term JUUL e-Cig exposure can increase the risk of thrombotic events [22]. To our knowledge, no study has yet addressed the effects of JUUL e-Cigs on the cerebrovascular system. In this study, we have investigated the impact of short-term JUUL e-Cig exposure on brain injury, BBB tight junction (TJ) proteins, and inflammatory and oxidative stress markers in ischemic stroke in direct comparison with TS.

## Results

### TS-exposed mice had higher plasma nicotine and cotinine level than JUUL-exposed mice

**Fig. 1:**
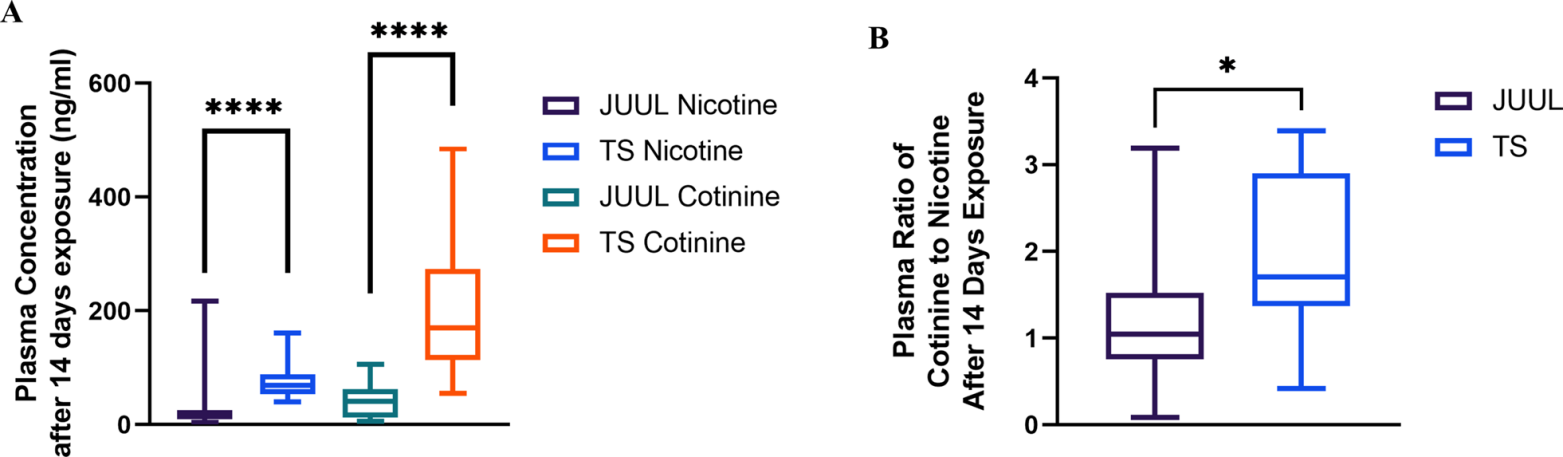
Plasma concentration and the ratio of nicotine and cotinine in mice after 14 days of JUUL and TS exposure. **A** Plasma nicotine and cotinine concentration after 14 days of JUUL and TS exposure. **B** Plasma ratio of cotinine to nicotine after 14 days of JUUL and TS exposure. ****P < 0.000; n = 23 for TS and n = 31animals for JUUL

Average plasma nicotine concentrations in JUUL and TS-exposed mice were 22.5 ± 6.68 ng/ml and 74.76 ± 5.95 ng/ml, respectively, whereas those of cotinine were 42.58 ± 5.2 ng/ml and 194.7 ± 24.42 ng/ml, respectively. Plasma concentration of nicotine was significantly higher in TS-exposed mice (P < 0.0001) compared to JUUL-exposed mice (Fig. 1A). Similarly, TS-exposed mice had a higher plasma level of cotinine (P < 0.0001) than JUUL-exposed mice (Fig. 1A). Further, when we measured the ratio of plasma cotinine to nicotine, we found that the ratio was significantly decreased in JUUL-exposed mice (1.25) (P < 0.05) than in TS-exposed mice (1.88) (Fig. 1B). This ratio characterizes nicotine metabolism to cotinine in mice after JUUL/TS exposure.

### JUUL and TS exposure caused weight reduction in mice, and TS exposure induced hyperactivity

**Fig. 2:**
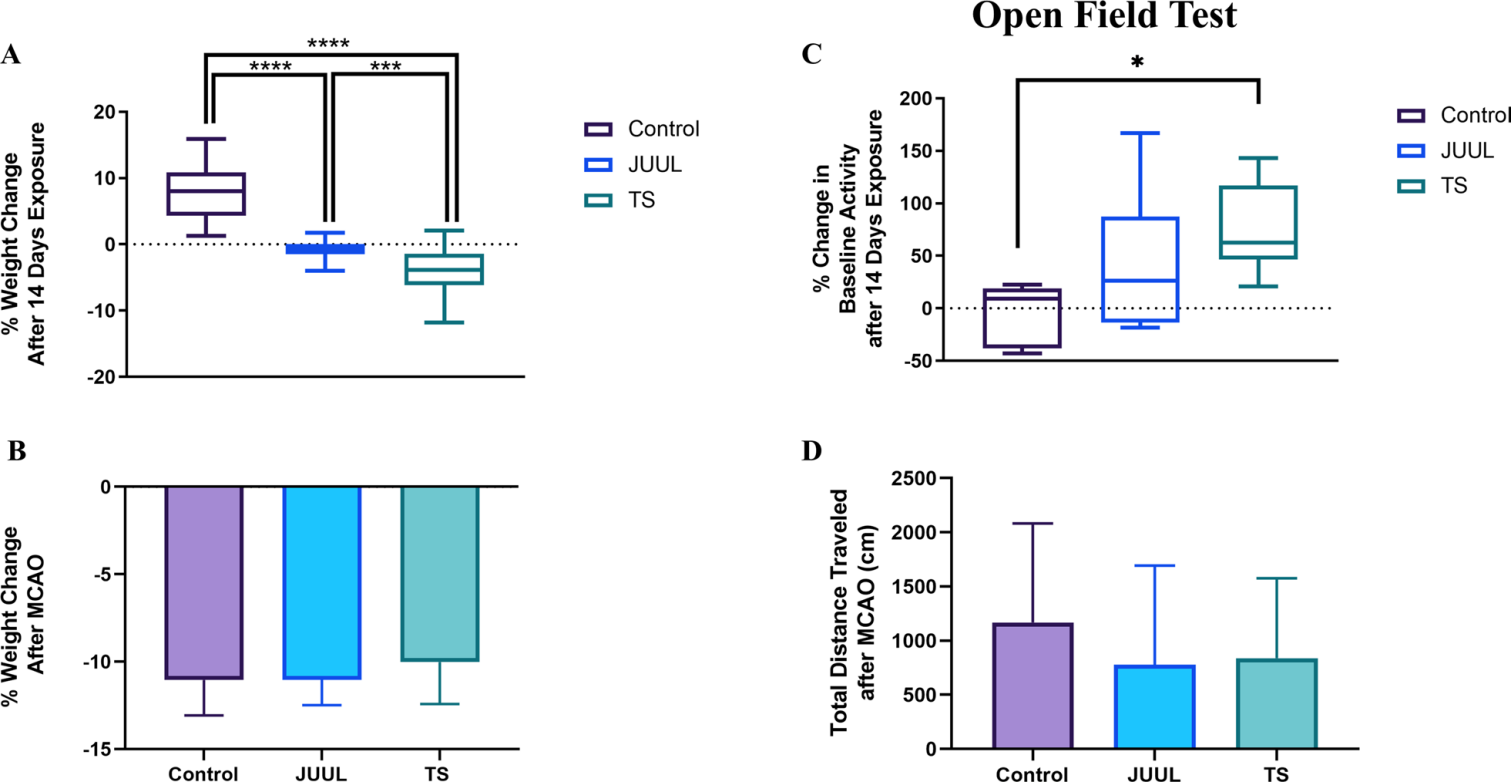
Weights and locomotor activity of mice after 14 days of JUUL and TS exposure. **A** Percent weight change in mice after 14 days of JUUL and TS exposure. **B** Percent weight change in JUUL/TS-exposed mice 24 h after MCAO. **C** Percent change in baseline locomotor activity in mice after 14 days of JUUL and TS exposure. **D** Total distance traveled (cm) by JUUL/TS-exposed mice 24 h after MCAO. *P < 0.05, ***P < 0.001, ****P < 0.0001; n = 38, 32, and 21 for control, JUUL, and TS respectively (**A**), n = 16, 16, and 13 for control, JUUL, and TS respectively (**B**), n = 6, 11, and 6 for control, JUUL, and TS respectively (**C**), n = 6 for each of the 3 groups (**D**)

We measured the weight of the mice after 14 days of JUUL or TS exposure and 24 h after MCAO (Fig. 2A, B). We found that JUUL (P < 0.0001) or TS (P < 0.0001) exposure for 14 days drastically reduced the weight of the mice compared to the control (Fig. 2A). TS-exposed mice also had significant weight reduction (P < 0.001) compared to JUUL-exposed mice. MCAO caused weight reduction in all groups, but no significant weight difference was observed among control, JUUL, and TS-exposed mice 24 h after MCAO. The locomotor activity of the mice was measured by open field test after 14 days of JUUL or TS exposure and 24 h after MCAO (Fig. 2C, D). JUUL-exposed mice had a non-significant increase in percent baseline activity change compared to control, while TS-exposed mice showed significant hyperactivity (P < 0.05) compared to control mice (Fig. 2C). No significant difference was observed in the total distance traveled by the mice 24 h after MCAO among the 3 groups (Fig. 2D). TS exposure significantly affected the weight and locomotor activity of the mice, while the effects of JUUL exposure were to a lesser extent.

### JUUL and TS-exposed mice had a worsening brain injury 24 h after MCAO

**Fig. 3:**
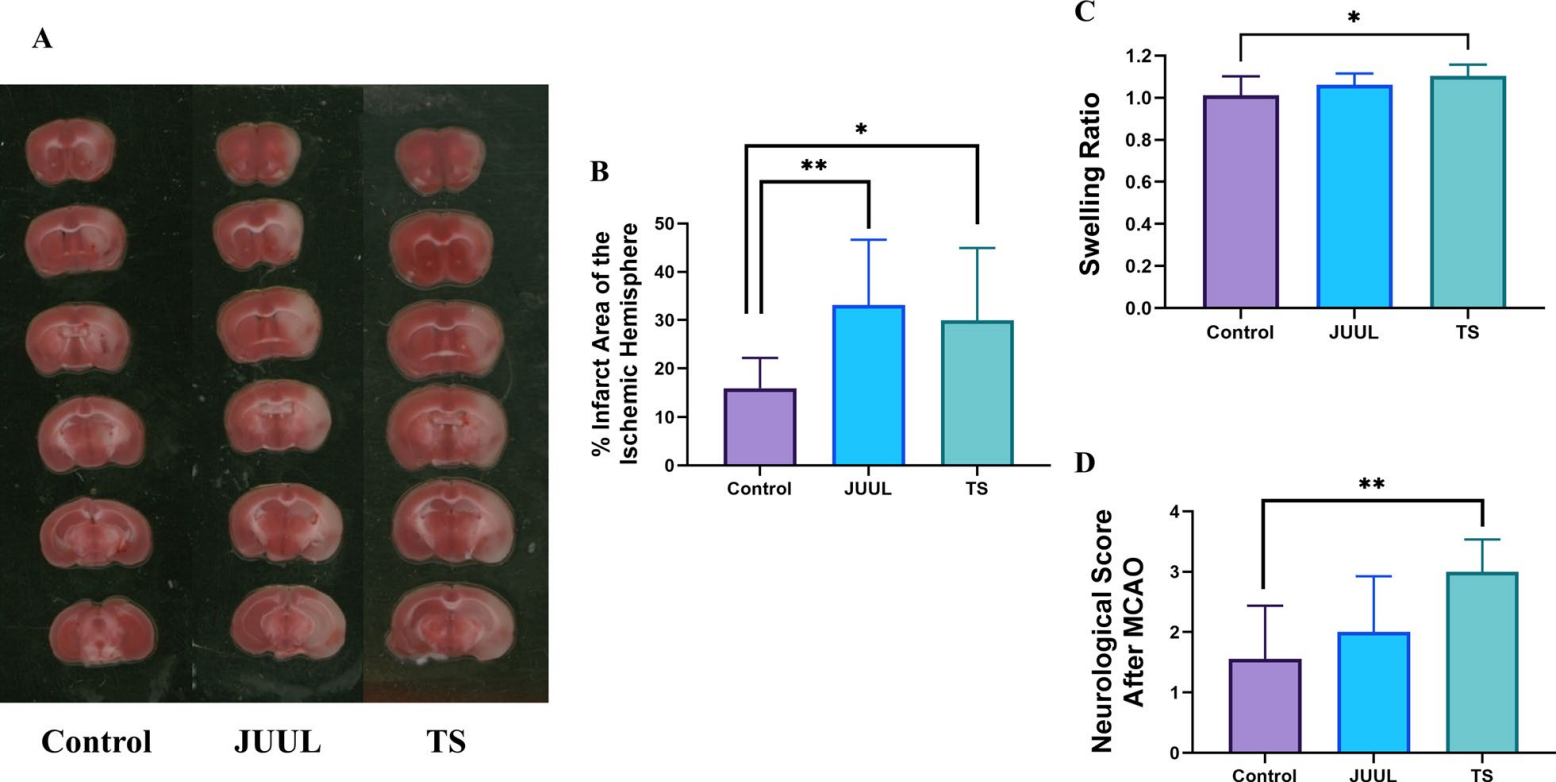
Brain injury, swelling ratio, and neurological score in mice 24 h after MCAO. **A** TTC staining of brain slices exposed to JUUL/TS. **B** Percent infarct area in the ischemic brain hemisphere of JUUL/TS-exposed mice. **C** The swelling ratio of JUUL/TS-exposed mice. **D** The neurological score of JUUL/TS-exposed mice. *P < 0.05, **P < 0.01; n = 12, 10, and 7 for control, JUUL, and TS respectively (**B**, **C**), n = 9, 8, and 8 for control, JUUL, and TS respectively (**D**)

We did 2,3,5-tripheyltetrazolium chloride (TTC) staining 24 h after MCAO to evaluate brain injury in the mice (Fig. 3A–C). JUUL (P < 0.01) and TS-exposed (P < 0.05) mice had significantly increased brain infarct area compared to control mice (Fig. 3A, B). There was also an increase in brain swelling ratio in TS-exposed mice (P < 0.05) compared to control (Fig. 3A, C). Further, the neurological score was significantly worsened in TS-exposed mice (P < 0.01 vs. control) 24 h after MCAO (Fig. 3A, D). Although JUUL exposure increased brain infarct area, it did not significantly affect brain swelling and neurological function after MCAO.

### Plasma IL-6 level was increased, and thrombomodulin level was decreased 24 h after MCAO

**Fig. 4:**
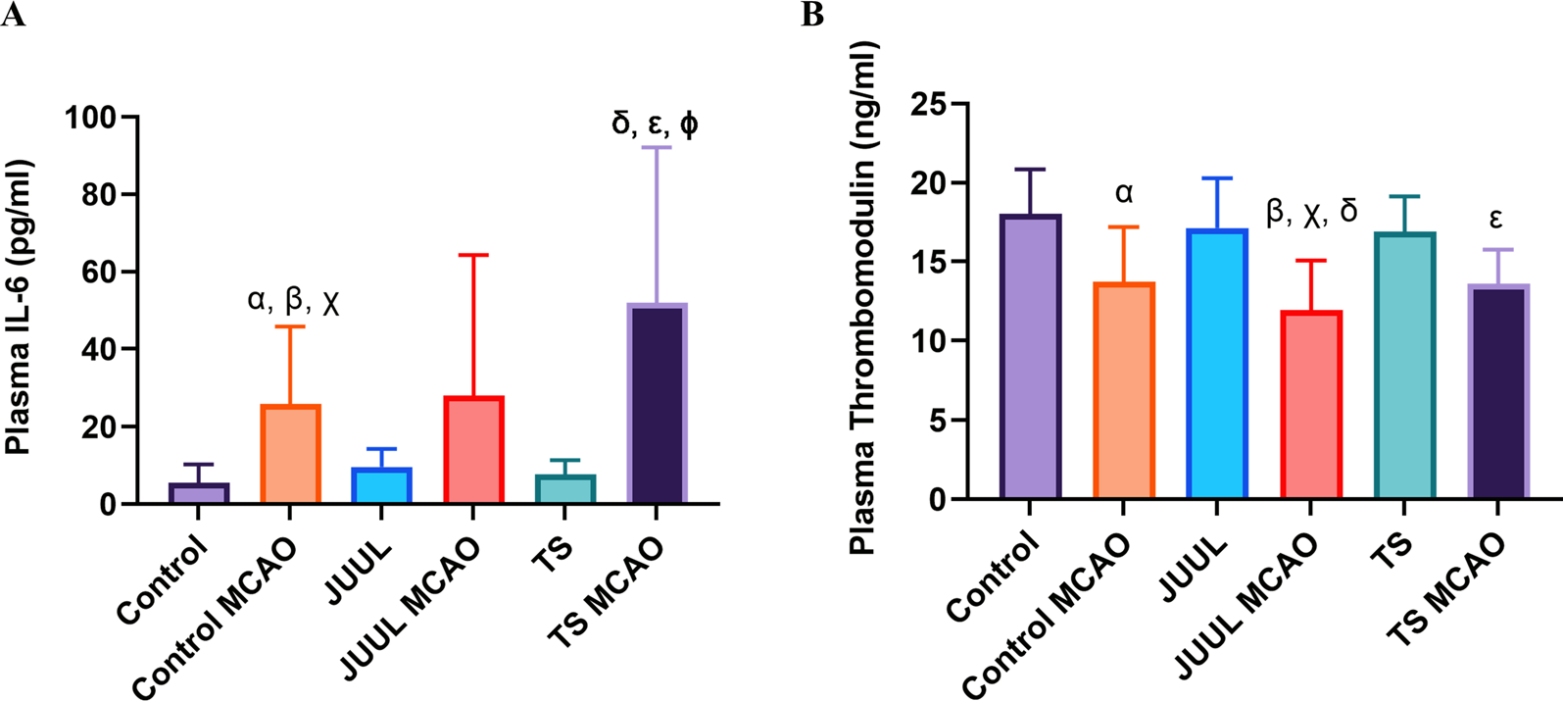
Plasma IL-6 and thrombomodulin levels in mice after 14 days of JUUL/TS exposure. **A** Plasma IL-6 concentration in mice after 14 days of JUUL and TS exposure with or without MCAO. **B** Plasma thrombomodulin concentration in mice after 14 days of JUUL and TS exposure with or without MCAO. For (**A**), α P < 0.01, β P < 0.05, and X P < 0.05 refers to control vs. control MCAO, JUUL vs. control MCAO, and TS vs. control MCAO, respectively, and δ P < 0.01, ε P < 0.05, and φ P < 0.01 refers to control vs. TS MCAO, JUUL vs. TS MCAO, and TS vs. TS MCAO, respectively. For (**B**), α P < 0.05 refers to control vs. control MCAO, β P < 0.0001, X P < 0.001, and δ P < 0.01 refers to control vs. JUUL MCAO, JUUL vs. JUUL MCAO, and TS vs. JUUL MCAO, respectively, and ε P < 0.05 refers to control vs. TS MCAO; n = 10, 9, and 8 for control, JUUL, and TS without MCAO groups respectively and n = 7, 9, and 5 for control, JUUL, and TS MCAO groups respectively (**A**), n = 15, 15, and 9 for control, JUUL, and TS without MCAO groups respectively and n = 8, 11, and 6 for control, JUUL, and TS MCAO groups respectively (**B**)

Plasma IL-6 and thrombomodulin levels were measured in mice by ELISA after 14 days of JUUL or TS exposure and 24 h after MCAO (Fig. 4A, B). IL-6 level, a key inflammatory marker, was higher in control MCAO (P < 0.01 vs. control, P < 0.05 vs. JUUL, and P < 0.05 vs. TS) and TS MCAO (P < 0.01 vs. control, P < 0.05 vs. JUUL, and P < 0.01 vs. TS) groups (Fig. 4A). In contrast, the IL-6 level in JUUL MCAO was not significant. In contrast, plasma thrombomodulin level was lower in control MCAO (P < 0.05 vs. control), JUUL MCAO (P < 0.0001 vs. control, P < 0.001 vs. JUUL, and P < 0.01 vs. TS), and TS MCAO (P < 0.05 vs. control) groups (Fig. 4B). JUUL MCAO had a more significant effect than TS MCAO for plasma thrombomodulin, indicating more coagulation potential.

### JUUL and TS exposure reduces TJ protein expression at the BBB 24 h after MCAO

**Fig. 5:**
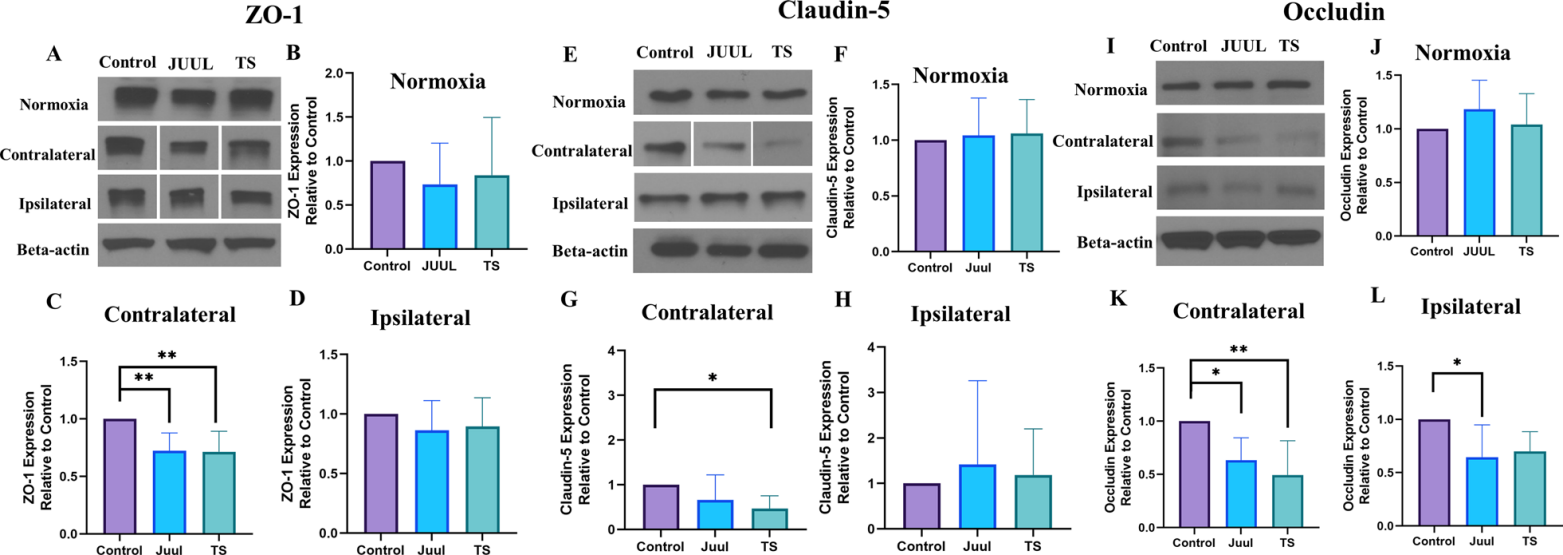
Brain ZO-1, claudin-5, and occludin expression in mice after 14 days of JUUL/TS exposure with or without MCAO. **A** Western blot images of ZO-1 expression in normoxic brain and contralateral and ipsilateral brain hemispheres 24 h after MCAO. **B**–**D** Quantification of brain ZO-1 expression normalized to beta-actin and expressed as relative to control (1.0) in normoxic, contralateral, and ipsilateral brain regions. **E** Western blot images of claudin-5 expression in normoxic brain and contralateral and ipsilateral brain hemispheres 24 h after MCAO. **F**–**H** Quantification of brain claudin-5 expression normalized to beta-actin and expressed as relative to control (1.0) in normoxic, contralateral, and ipsilateral brain regions, respectively. **I** Western blot images of occludin expression in normoxic brain and contralateral & ipsilateral brain hemispheres 24 h after MCAO. **J–L** Quantification of brain occludin expression normalized to beta-actin and expressed as relative to control (1.0) in normoxic, contralateral, and ipsilateral brain regions. *P < 0.05, ***P < 0.001; n = 8 for each group (**B**), n = 6, 6, and 5 for control, JUUL, and TS respectively (**C**), n = 7, 7, and 6 for control, JUUL, and TS respectively (**D**), n = 9 for each group (**F**), n = 8, 7, and 6 for control, JUUL, and TS respectively (**G**), n = 8, 8, and 6 for control, JUUL, and TS respectively (**H**), n = 9 for each group (**J**), n = 7, 6, and 5 for control, JUUL, and TS respectively (**K**), n = 6 for each group (**L**). Cropped images from blots have been used in some cases to improve the clarity and conciseness of the presentation. The cropped images are delineated with white space; full-length blots developed by X-ray films are presented in Additional file 1: Fig. S1

**Fig. 6:**
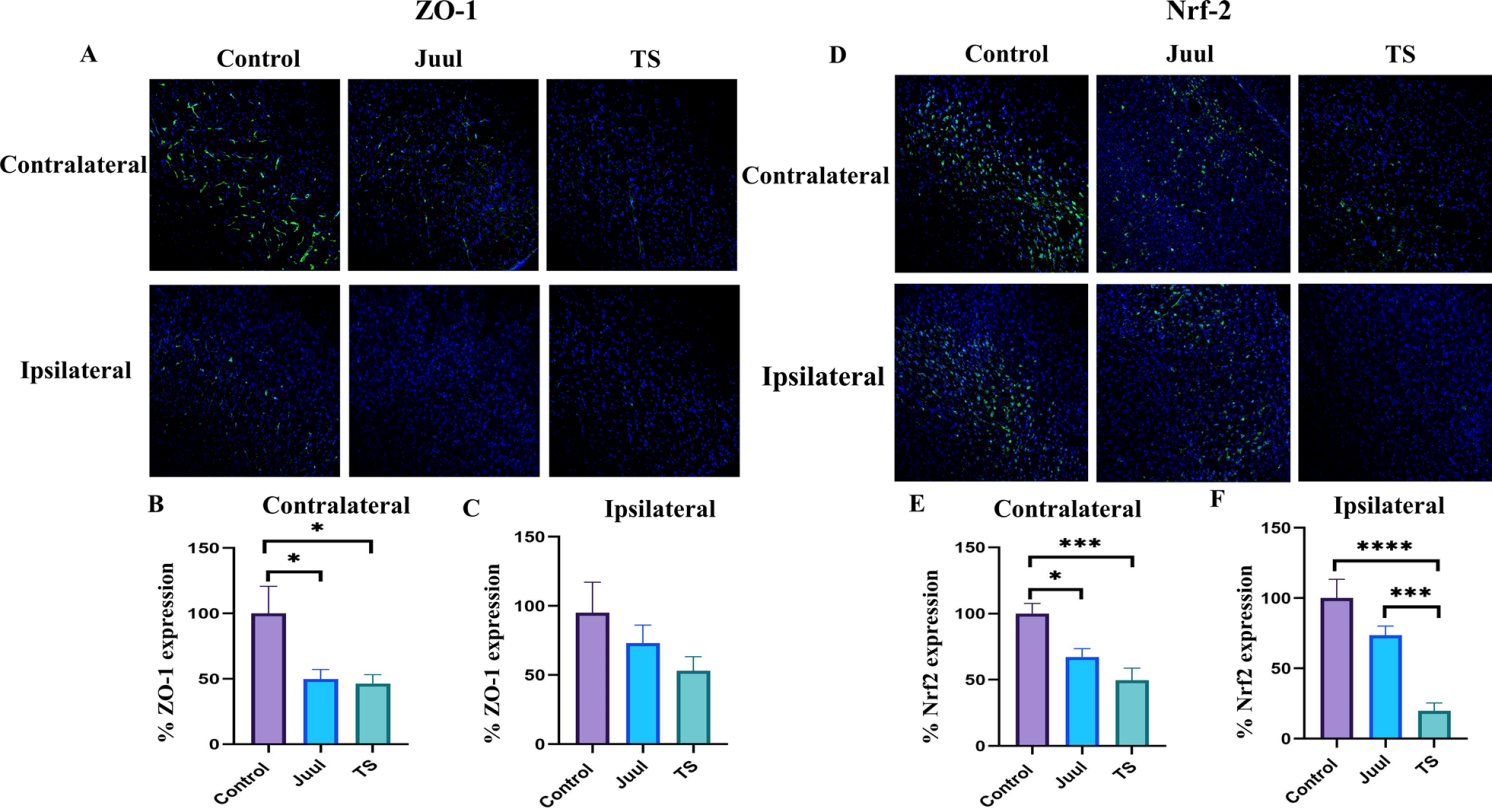
Immunofluorescence studies showed brain ZO-1 and Nrf2 expression in mice after 14 days of JUUL/TS exposure with MCAO. **A** Immunofluorescence images of brain slices where green color represents ZO-1 while blue color represents DAPI (nuclear marker). Quantification of contralateral (**B**) and ipsilateral (**C**) ZO-1 expression relative to DAPI, expressed as a percentage. **D** Immunofluorescence images of brain slices where green color represents Nrf2 while blue color represents DAPI (nuclear marker). Quantification of contralateral (**E**) and ipsilateral (**F**) Nrf2 expression relative to DAPI, expressed as a percentage. Contralateral, non-injured hemisphere of the stroke brain; ipsilateral, injured hemisphere of the stroke brain. *P < 0.05, ***P < 0.001, ****P < 0.0001; n = 4 mice for each group

**Fig. 7:**
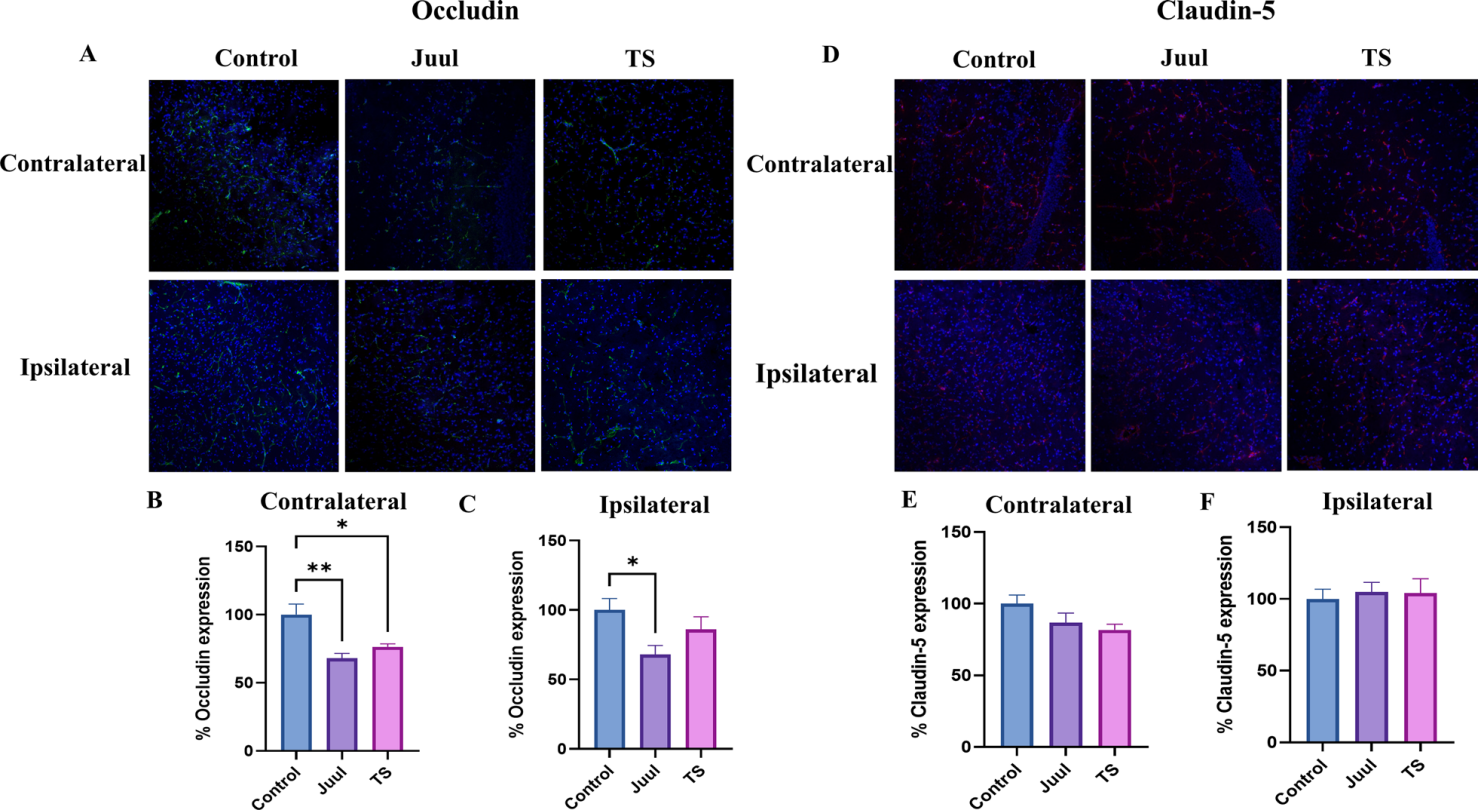
Immunofluorescence studies showing brain occludin and claudin-5 expression in mice after 14 days of JUUL/TS exposure and MCAO. **A** Immunofluorescence images of brain slices where green color represents occludin while blue color represents DAPI (nuclear marker). Quantification of contralateral (**B**) and ipsilateral (**C**) occludin expression relative to DAPI, expressed as a percentage. **D** Immunofluorescence images of brain slices where red color represents claudin-5 while blue color represents DAPI (nuclear marker). Quantification of contralateral (**E**) and ipsilateral (**F**) claudin-5 expression relative to DAPI, expressed as a percentage. Contralateral, non-injured hemisphere of the stroke brain; ipsilateral, injured hemisphere of the stroke brain. *P < 0.05, **P < 0.01; n = 4 mice for each group

We measured the expression of BBB tight junction (TJ) proteins (claudin-5 and occludin) and TJ-associated protein ZO-1 by western blot and immunofluorescence in the normoxic and contralateral and ipsilateral brain hemispheres 24 h after MCAO (Figs. 5A–L, 6A–C, 7A–F). JUUL and TS exposure had a comparable damaging effect on the TJ-associated protein ZO-1. JUUL (P < 0.01) and TS (P < 0.01)- exposed mice had a significant reduction of ZO-1 expression after MCAO in the contralateral brain hemisphere compared to control (Fig. 5A, C) in the western blot. There was no substantial change in normoxic and ipsilateral brain ZO-1 levels among the groups (Fig. 5A, B, D). This data is supported by immunofluorescence studies which showed JUUL (P < 0.05) and TS (P < 0.05)-exposure reduces ZO-1 expression in the contralateral brain hemisphere (Fig. 6A, B) compared to control while there was a non-significant downregulation of ZO-1 in the ipsilateral hemisphere (Fig. 6A, C). In the western blot, claudin-5 expression in the contralateral brain hemisphere of TS-exposed mice was reduced (P < 0.05) compared to control (Fig. 5E, G), while JUUL exposure had no significant effect. Also, no significant change in claudin-5 expression in the normoxic and ipsilateral brain regions was observed among the groups (Fig. 5E, F, H). In immunofluorescence studies, claudin-5 expression showed a decreasing trend in the contralateral hemisphere with JUUL and TS exposure, but it was not statistically significant (Fig. 7D, E). No change in claudin-5 expression was observed in the ipsilateral hemisphere (Fig. 7D, F). Occludin expression was also significantly reduced in the contralateral brain regions of JUUL (P < 0.05 vs. control) and TS (P < 0.01 vs. control)-exposed mice (Fig. 5I, K) in western blot experiments. Further, JUUL-exposed mice had decreased occludin expression (P < 0.05) in the ipsilateral brain region compared to control (Fig. 5I, L), with no significant change observed with TS exposure. Also, no change in occludin expression in the normoxic brains was observed (Fig. 5I, J). Immunofluorescence studies strongly support these results, where JUUL (P < 0.01) and TS (P < 0.05) exposure decreased occludin expression in the contralateral hemisphere (Fig. 7A, B). In the ipsilateral hemisphere, only JUUL exposure (P < 0.05) decreased occludin expression (Fig. 7A, C). JUUL exposure had a more significant effect on occludin expression in the ischemic brain than TS.

### JUUL and TS exposure decreases Nrf2, and TS exposure increases ICAM-1 expression in the brain 24 h after MCAO

**Fig. 8:**
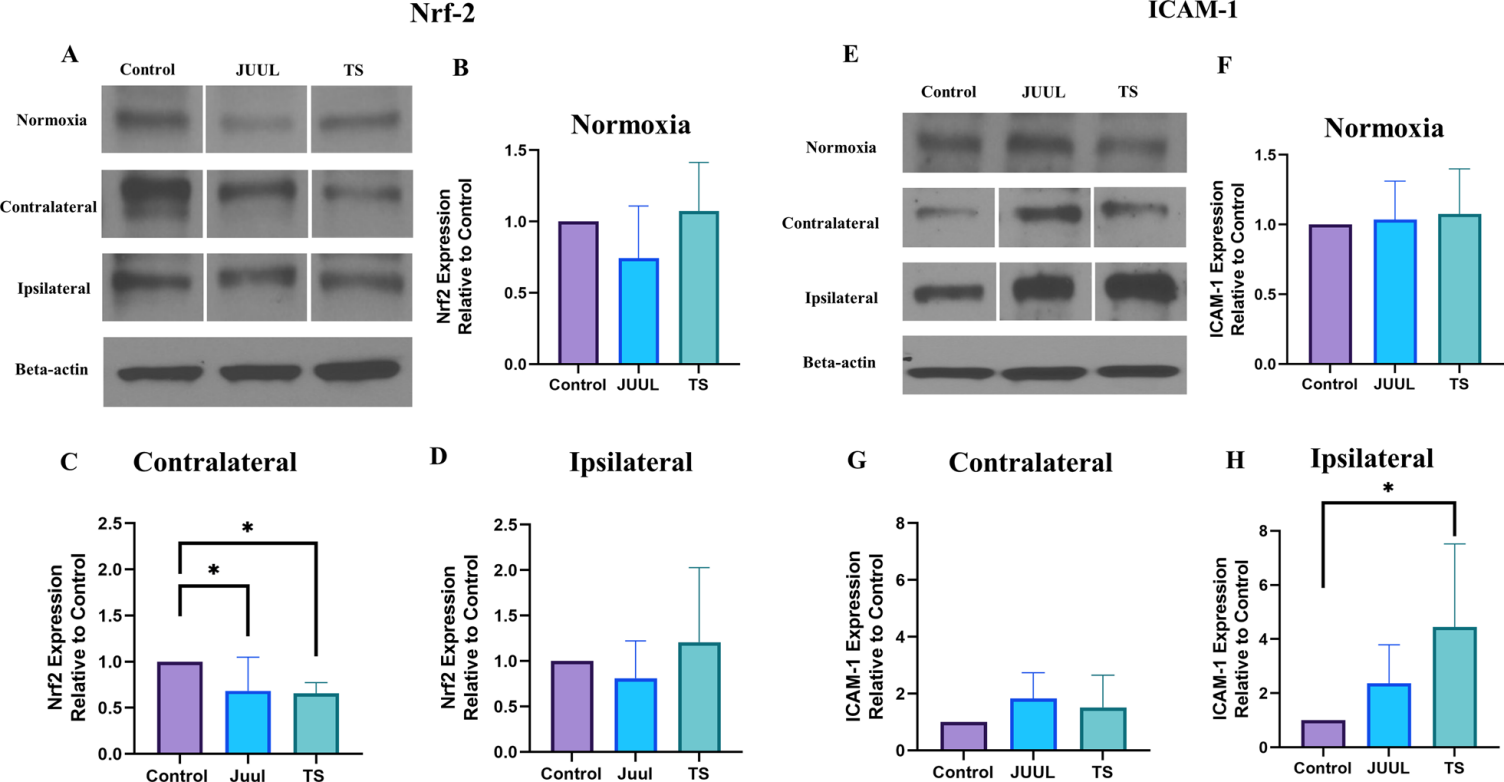
Brain Nrf2 and ICAM-1 expression in mice after 14 days of JUUL/TS exposure with or without MCAO. **A** Western blot images of Nrf2 expression in normoxic brain and contralateral & ipsilateral brain hemispheres 24 h after MCAO. **B**–**D** Quantification of brain Nrf2 expression normalized to beta-actin and expressed as relative to control (1.0) in normoxic, contralateral, and ipsilateral brain regions. **E** Western blot images of ICAM-1 expression in normoxic brain and contralateral & ipsilateral brain hemispheres 24 h after MCAO. **F**–**H** Quantification of brain ICAM-1 expression normalized to beta-actin and expressed as relative to control (1.0) in normoxic, contralateral, and ipsilateral brain regions. *P < 0.05; n = 9 for each group (B), n = 7, 7, and 6 for control, JUUL, and TS respectively (**C**, **D**), n = 9 for each group (**F**), n = 6, 5, and 5 for control, JUUL, and TS respectively (**G**, **H**). Cropped images from blots have been used in some cases to improve the clarity and conciseness of the presentation. The cropped images are delineated with white space; full-length blots developed by X-ray films are presented in Additional file 1: Fig. S1

We then measured brain expression of the inflammatory marker ICAM-1 and the antioxidant marker Nrf2 by western blot in the normoxic and, contralateral & ipsilateral brain hemispheres 24 h after MCAO (Fig. 8A–H). No significant change was observed in normoxic brain Nrf2 expression among the groups (Fig. 8A, B). JUUL (P < 0.05) and TS (P < 0.05)-exposed mice had a significant reduction of Nrf2 expression after MCAO in the contralateral brain hemisphere compared to control (Fig. 8A, C). Interestingly, immunofluorescence studies supported the western blot findings in the contralateral hemisphere, showing a significant reduction of Nrf2 expression by JUUL (P < 0.05) and TS (P < 0.001) exposure (Fig. 6D, E). No significant difference was observed in ipsilateral Nrf2 expression by western blot (Fig. 8A, D). However, immunofluorescent studies showed a significant reduction of Nrf2 by TS (P < 0.0001) and JUUL (P < 0.001) exposure in the ipsilateral hemisphere (Fig. 6D, F). Western blot results showed that ICAM-1 expression was significantly increased in the ipsilateral brain regions of TS (P < 0.05 vs. control)-exposed mice (Fig. 8E, H). There was no significant change in ICAM-1 expression in the group’s normoxic and contralateral brain regions (Fig. 8E–G).

## Discussion

JUUL e-Cigs have become extremely popular recently, and studies are needed to elucidate their possible toxic effects on the cerebrovascular system. In this study, we have investigated the impact of short-term JUUL exposure on ischemic brain injury, BBB TJ proteins, and inflammatory and antioxidative markers compared with TS in mice. To our knowledge, this is the first study that evaluated cerebrovascular toxicities of JUUL with a side-by-side comparison with TS using a preclinical model of ischemic stroke.

We have used a well-established smoking/vaping exposure model for this study [12, 20, 23, 24]. Plasma nicotine and cotinine level in mice after 2 weeks of TS and JUUL exposure were comparable to previously published in vivo studies [23, 25–27]. Importantly, these concentrations were also reflective of human cigarette smokers [23, 28]. We found higher plasma nicotine and cotinine concentrations in TS-exposed mice than JUUL-exposed mice, consistent with published studies in our laboratory and others involving TS and e-Cigs [23, 25–27]. Further, nicotine to cotinine metabolism was also reduced in plasma of JUUL-exposed mice than that of TS. One possible explanation of this could be the formation of nicotyrine by the gradual oxidation of e-liquids exposed to air. Nicotyrine inhibits CYP2A enzymes in the lungs and liver, thus could inhibit nicotine metabolism to cotinine by CYP2A6 [29].

The weights of the mice were drastically reduced after two weeks of TS exposure. Significant weight reduction was also observed with JUUL exposure. It has been widely reported that nicotine and TS can reduce body weight in preclinical [30, 31] and clinical studies [32, 33]. Vaping was also shown to decrease body weight [34, 35]. In our study, TS-exposed mice showed hyperactivity in the open field test. It is consistent with other studies which showed that short-term TS exposure increases physical activity in rodents compared to control [36]. Interestingly, mice exposed to long-term (10 months) of TS [36] or heavy human smokers [37, 38] displayed reduced physical activity, suggesting a differential effect induced by acute vs. chronic nicotine exposure.

Our study found that both JUUL and TS can increase brain injury after ischemic stroke. TS exposure also worsened brain swelling and neurological functions. This result is consistent with our group's previous study, which showed that TS and e-Cig (Blu) exposure [12] could worsen ischemic brain injury. We also showed that acute administration of nicotine and nicotine-containing TS extract increases brain edema/swelling and infarct ratio after MCAO [13]. Other researchers have demonstrated that exposure to nicotine or TS can worsen ischemic brain damage in rodents [19, 39, 40].

JUUL or TS exposure did not cause any significant change in our study's plasma concentration of the inflammatory marker IL-6. However, we found that ischemic stroke increases the plasma level of IL-6. Plasma IL-6 concentration 24 h after ischemic stroke was the highest in TS pre-exposed mice, although no significant difference was found among the groups. Per our findings, IL-6 has been identified as a prognostic marker for ischemic stroke, as it was correlated with worsened ischemic brain injury and outcome in clinical [41–44] and preclinical [45, 46] studies. Thrombomodulin is a natural anticoagulant [47], which exerts a protective effect in acute ischemic stroke by inhibiting coagulation, fibrinolysis, and inflammation, stabilizing barrier function, and increasing blood flow [48]. We found decreased plasma thrombomodulin concentrations after ischemic stroke, but no significant effects of JUUL or TS- pre-exposure were observed after MCAO. The serum concentration of soluble thrombomodulin decreased at the acute stage and increased after six months of ischemic stroke onset, as shown in a clinical study [49]. In contrast, plasma thrombomodulin was higher in a clinical study by Zhang et al., which could be due to a small sample number [50]. In another study, expression of endothelial thrombomodulin was decreased in the ischemic core region but increased in the peri-infarct area, compared to the contralateral side [51].

Disruption of the BBB is one of the key pathophysiological features of ischemic stroke, contributing to ischemic brain injury and neurological disturbances [52]. Ischemic stroke causes disruptions in the TJ proteins at the BBB [53]. Claudin-5 is a crucial BBB TJ protein responsible for increased paracellular permeability in experimental stroke settings if disrupted [52, 54]. Occludin regulates functional integrity and paracellular permeability of the BBB [55, 56], while ZO-1 connects transmembrane TJ proteins to the actin cytoskeleton [57]. TS and e-Cig exposure decreased the expression of ZO-1 in an in vitro model of BBB [12]. Prasad et al. found no significant change in ZO-1 and occludin expression after 2 weeks of TS exposure. However, 4 weeks of TS exposure decreased the expression of those TJ proteins [58]. Our study did not observe any significant change in the expression of the TJ proteins (ZO-1, claudin-5, and occludin) after two weeks of JUUL or TS exposure. This could be due to the inherent difference between in vitro and in vivo systems and the amount & duration of exposure. Our western blot and immunofluorescence studies showed reduced ZO-1 and occludin in the contralateral hemisphere by JUUL and TS exposure.

Interestingly, only JUUL exposure reduced occludin expression in the ipsilateral hemisphere. Claudin-5 expression was not substantially affected by JUUL and TS exposure, as observed in our study. We observed the harmful effects of JUUL or TS on BBB TJ protein expression only after ischemic stroke, which implies that adding another insult accentuates the harmful effects caused by JUUL or TS exposure on the BBB. Studies investigating the impact of TS and/or e-Cig on BBB and TJ proteins in acute ischemic stroke have been scarce. Acute exposure to TS extract worsened BBB disruption after the ischemia-like condition in an in vitro study [59]. Sladojevic et al. showed that claudin-5 expression in the ipsilateral brain hemisphere was decreased after MCAO, but no change of this protein in the contralateral brain hemisphere was observed [60].

Similarly, ZO-1 and occludin expression in the ischemic cortex was significantly decreased in a photothrombotic stroke model; however, their expression was unchanged in the contralateral hemisphere [53]. By contrast, researchers found BBB damage in the contralateral brain hemisphere in an in vivo model of sub-acute ischemic stroke [61]. This change in the contralateral brain was associated with reactive astrocytes and microglia in that hemisphere, indicating an inflammatory response [61]. Interestingly, significant changes in brain activity and functional connectivity in the contralateral brain hemisphere in acute ischemic stroke have been reported, linked with functional recovery [62]. The reduction of TJ proteins' expression in the contralateral hemisphere by TS or JUUL pre-exposure, as observed in our study, could be due to an enhanced release of inflammatory mediators (cytokines, chemokines, matrix metalloproteinases—MMPs, and vascular endothelial growth factor—VEGF) in the bloodstream from the ischemic hemisphere, which may create a profound effect on the non-ischemic hemisphere. The mechanisms of these observed changes will be the subject of future investigation, with focused experiments measuring astroglia and microglia markers in both hemispheres. Overall, these findings bear significance as by reducing the otherwise unchanged TJ proteins in the contralateral hemisphere, the whole ischemic brain could be indirectly affected, leading to worsened brain damage after acute ischemic stroke. One limitation of the current study is that we have measured TJ protein expression at the BBB with western blot using total brain tissue instead of studying the expression of those proteins in isolated brain microvessels. Therefore, the reported values may slightly differ from TJs proteins expression levels directly measured from purified brain microvessels. However, this procedure has been previously used for similar studies [12, 24, 58] to assess the cerebrovascular impact of smoking on BBB TJs expression under diseased conditions (e.g., traumatic brain injury—TBI) and the protective effect of potential countermeasures.

Oxidative stress and inflammation play a vital role in the pathobiology of ischemic stroke. Nrf2 is a nuclear transcription factor regulating the cellular antioxidative response system. Nrf2 has also been shown to play an essential role in TS-mediated BBB toxicity and ischemic stroke. Nrf2 was previously shown to be downregulated by chronic TS and/or e-Cig exposure in vitro and/or in vivo [12, 58]. However, we did not observe any significant change after 2 weeks of JUUL or TS exposure. Nrf2 was upregulated in tMCAO studies [63] and also exerted protective effects against ischemic brain damage [63, 64]. Dang et al. demonstrated cellular expression of Nrf2 in ischemic rat brains by double immunofluorescence staining [65]. They found enhanced Nrf2 expression in the ipsilateral penumbra region in both neurons and glial cells (astrocytes, microglia). However, Nrf2 was significantly induced only in neurons in the contralateral brain hemisphere. In our western blot studies, Nrf2 expression was significantly decreased in the contralateral brain after MCAO with JUUL or TS pre-exposure. In immunofluorescence studies, we found a similar reduction of Nrf2 in the contralateral brain by JUUL or TS exposure. However, immunofluorescence studies also showed a significant reduction of Nrf2 in the ipsilateral brain regions by JUUL or TS exposure, contrasting the western blot results. Using whole brain tissue for western blot may have contributed to the contradictory results. Kaisar et al. also observed that e-Cig or TS exposure decreases brain Nrf2 expression after ischemic stroke [12].

By reducing the antioxidative and cytoprotective actions of Nrf2, JUUL and TS could worsen the ischemic brain damage and neurological outcome. ICAM-1 is an inflammatory marker that helps in leukocyte infiltration in response to an ischemic event [66]. We did not observe any significant change in ICAM-1 expression after JUUL or TS exposure. Prasad et al. also observed no significant change in ICAM-1 expression after 2 weeks of TS exposure [58]. Contrastingly, TS extract increased the expression of ICAM-1 in hCMEC/D3 BBB endothelial cells [67]. In another study, 2 weeks of TS and e-Cig exposure increased brain ICAM-1 expression [12]. Higher endothelial ICAM-1 expression was observed in the brain after acute ischemic stroke in clinical [66, 68, 69], and preclinical studies [69–71]. In our study, ICAM-1 was significantly increased in the ischemic brain hemisphere of TS pre-exposed mice, but not in JUUL-exposed mice. This increase in inflammation could be one of the mechanisms of TS-mediated exacerbated ischemic brain injury and neurological damage. The unchanged ICAM-1 expression after only JUUL or TS exposure could be due to differences between in vitro and in vivo systems and duration of exposure, as explained earlier. The increase of ICAM-1 by TS exposure in the ipsilateral brain can also be explained by the observed decrease of Nrf2 in the same region in immunofluorescent studies. Nrf2 and its downstream pathway exert protective effects against inflammation by regulating anti-inflammatory gene expression and inhibiting inflammation [72]. Overexpression of Nrf2 has been shown to inhibit TNF-α-induced ICAM-1 expression in human retinal pigment epithelial cells treated with lycopene [73]. On the other hand, knockdown of Nrf2 enhanced brain ICAM-1 expression in a mouse model of traumatic brain injury [74]. This inhibitory role of Nrf2 on ICAM-1 can explain the overexpression of the latter in the ischemic brain.

TS exerted more cerebrovascular toxicity than JUUL, as observed in some of our abovementioned findings, which could be due to the higher nicotine concentration in TS-exposed mice. Further, TS has thousands of toxic chemicals, which could also be responsible for the enhanced toxicities. In the future, we would like to investigate the cerebrovascular effects of 4 weeks of JUUL e-Cig exposure with higher nicotine (5%) concentration and compare it to TS exposure.

## Conclusion

This study found that short-term JUUL e-Cig exposure can cause harmful effects on ischemic brain injury and TJ protein expression at the BBB, although to a lesser extent than that of TS exposure. Only TS exposure caused hyperactivity in mice and altered our study's expression of brain inflammatory and oxidative stress markers after ischemic stroke. However, JUUL exposure caused more reduction of the TJ protein occludin than TS. In summary, short-term JUUL e-Cig or TS exposure can enhance the sensitivity to ischemic stroke injury by disrupting the expression of TJ proteins at the BBB.

## Materials and methods

### Animals and surgical procedures

All studies were approved by the IACUC of Texas Tech University Health Sciences Center, Lubbock, Texas (IACUC protocol# 20026). All experiments were performed in accordance with relevant guidelines and regulations. This study was not pre-registered, and no randomization/blinding was performed. Male C57BL/6 mice (Charles River Laboratories, Inc., Wilmington, MA) were kept under standardized light and dark conditions (12 h), humidity (70%), and temperature (22 °C). They were given ad libitum access to food and water.

**Fig. 9:**
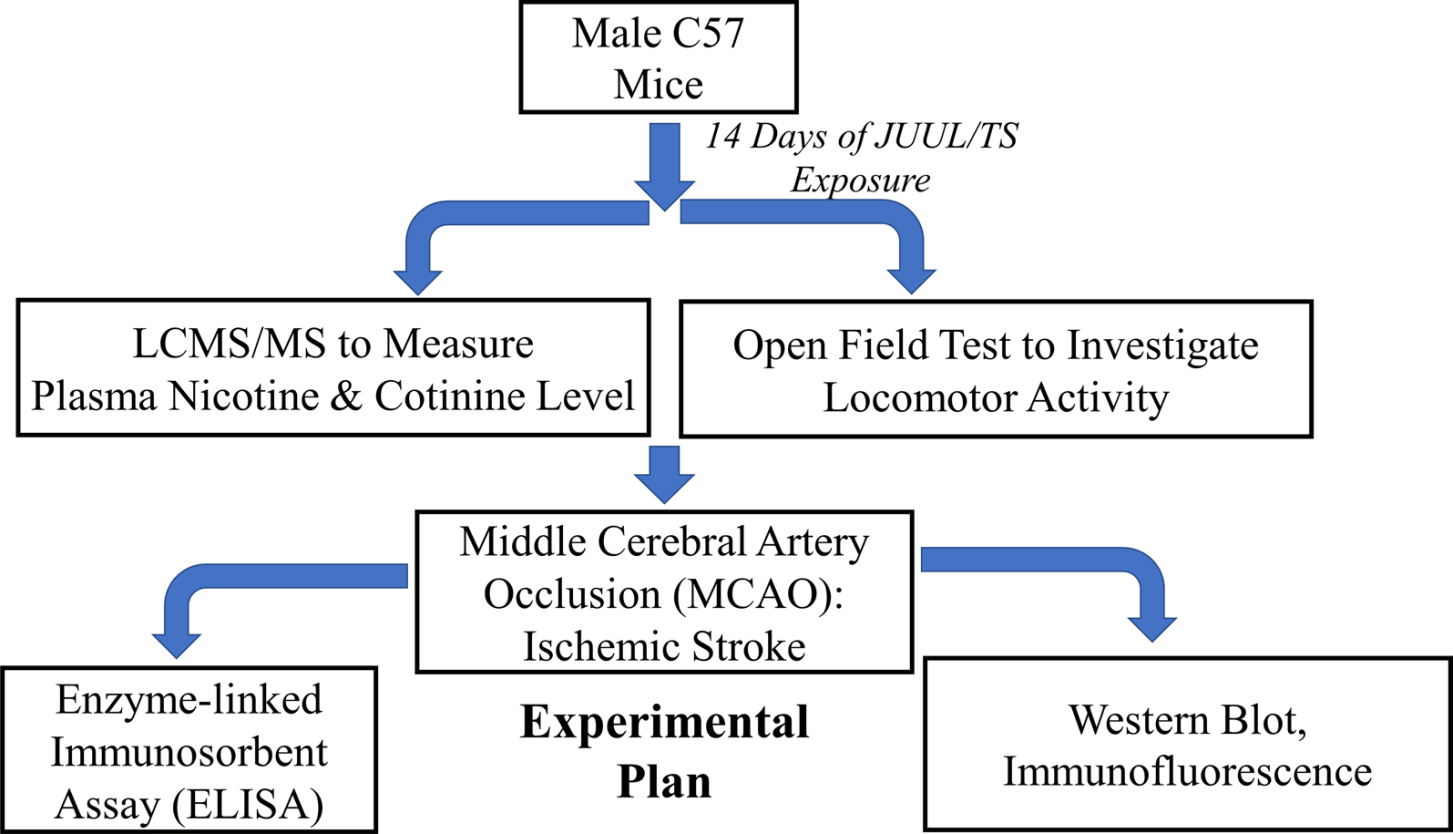
Flow diagram of the study design. Male C57 mice were exposed to JUUL or TS for 14 days. At the end of the exposure, plasma nicotine and cotinine level were measured by LCMS/MS, and an open field test was done to investigate the locomotor activity of the mice. Ischemic stroke was induced in mice by middle cerebral artery occlusion (MCAO) followed by reperfusion. 24 h after reperfusion, an enzyme-linked immunosorbent assay (ELISA) was performed to measure plasma IL-6 and thrombomodulin. Western blot and immunofluorescence were done to measure the expression of BBB tight junction proteins, and inflammatory and antioxidant markers in the brain

The behavior of the animals was monitored every day to minimize animal suffering. We applied the following exclusion criteria: severe weight loss, infections, or significant behavioral deficits (decreased mobility, seizures, lethargy). No animal was excluded from this study. The study is reported following ARRIVE (Animal Research: Reporting of In Vivo Experiments) guidelines. The research design is depicted as a flow diagram in Fig. 9.

### In vivo* TS/e-Cig vaping*

Mice were exposed (via direct inhalation) to JUUL e-Cig vapor (30 mg/ml nicotine) or 3R4F standardized research cigarettes (9.4 mg tar and 0.726 mg nicotine/cigarette equivalent to full flavor commercial products) mixed with oxygenated air or oxygenated air alone, 6 cycles/day for 14 days. A modified CORESTA (Cooperation Centre for Scientific Research Relative to Tobacco) standard smoking protocol adapted to study JUUL exposure (27.5 ml puff depth volume, 3 s puff duration, 2 puffs per 60 s, 32 puffs/cycle) and a modified CIR (Canadian Intense Regimen) standard smoking protocol (27.5 ml puff depth volume, 2 s puff duration, 2 puffs per 60 s, 32 puffs/cycle) to study TS exposure were followed in the laboratory. E-Cig vapor/TS was generated using a Single Cigarette Smoking Machines (SCSM, CH Technologies Inc., Westwood, NJ, USA) following a previously published method [12, 15]. These methods were followed to mimic the smoking behavior of a human chronic and heavy smoker/vaper and yield plasma levels of cotinine (43 and 195 ng/ml for JUUL and TS, respectively) which is in the range of blood cotinine levels found in other preclinical models of chronic TS/e-Cig exposure [23, 27, 34]. The smoking exposure was done between 9 am to 2 pm.

### Plasma nicotine and cotinine level measurement by LCMS/MS

The plasma concentration of nicotine and its principal metabolite cotinine were measured from the 14 days JUUL/TS-exposed mice by LCMS/MS analysis using Cotinine-d3 (MilliporeSigma, St. Louis, MO, USA) as internal standard (IS) following a previously published method [23]. In brief, samples were prepared by protein precipitation of 25 µl mouse plasma using acetonitrile at a 1:8 ratio. The mass spectrometer was operated in positive polarity under the multiple reaction monitoring mode using the electrospray ionization technique. The transitions of m/z 163.2 → 132.1, 177.2 → 98.0, and 180.2 → 101.2 were used to measure the nicotine, cotinine, and IS, respectively. The elution of nicotine (MilliporeSigma), cotinine (MilliporeSigma), and IS were at 1.89, 1.77, and 1.76 min, respectively. This was achieved with a mobile gradient phase consisting of 5 mM ammonium bicarbonate, acetonitrile, and methanol (3:1, v/v) at a 0.3 ml/min flow rate on a Kinetex EVO C18 column (Phenomenex, Torrance, CA, USA).

### Open field test

Open field test was performed according to our previously published study [20, 75]. This test evaluated the locomotor activity of the JUUL/TS-exposed or control mice with or without stroke. We used Versamax software (Accuscan Instruments., Columbus, OH) to automatically calculate the activity of the animals (total distance traveled). Briefly, mice were introduced to a 16″ × 16″ unobstructed glass chamber. They were monitored and recorded for 1 h. The first 10 min of 1 h was excluded as the acclimatization period. All experiments were performed between 8 am, and 11 am.

### Transient middle cerebral artery occlusion with reperfusion

Transient Middle cerebral artery occlusion (tMCAO) surgery was performed in mice (24–28 g) as previously reported [75, 76], using a Zeiss OP pico I surgical microscope (Carl Zeiss GmbH, Jena, Germany). Mice were anesthetized with 4% and maintained at 1.5% isoflurane in N_2_O/O_2_ mixture (70/30) using a SurgiVet Vaporizer (Smith Medical North America, Waukesha, WI, USA). Continuous blood flow was measured with a Laser Doppler probe (Moor Instruments, Wilmington, DE, USA). The probe was placed on the skull directly above the left MCA region (1 mm posterior and 3 mm lateral to the Bregma). Body temperature was maintained at 37 °C and controlled by a thermostatic blanket (TC-1000 Temperature Controller, CWE, USA). After aseptic preparation with betadine, a 1.5 cm long incision was made on the neck midline. The left common carotid artery (CCA), external carotid artery (ECA), and internal carotid artery (ICA) were carefully isolated from surrounding tissue. After CCA was occluded, a micro clip was placed on the ICA, and the ECA was ligated and coagulated. A small incision on the ECA was made to introduce a 6–0 nylon microfilament with a round tip (0.20–0.25 mm), and it was gradually inserted until it blocked the MCA bifurcation. A decrease in blood flow of 80% from baseline was considered a successful occlusion. After 30 min of occlusion, the nylon filament was carefully removed to restore blood flow, leading to reperfusion. An increase of 70% or more of the blood flow during occlusion was considered successful reperfusion.

### Neurological score

The neurological score was evaluated in mice 24 h after reperfusion. A four-point scale was utilized for this purpose [77]. A score of 0 indicated no neurological deficits, 1 indicated mild focal neurological deficit (animal showed forelimb flexion), 2 indicated moderate focal deficit (decreased resistance to lateral push and forelimb flexion), while 3 indicated severe focal deficit (animal showed all previous deficits plus circling).

#### 2,3,5-tripheyltetrazolium chloride staining

2,3,5-tripheyltetrazolium chloride (TTC) staining was used to demarcate viable brain tissue after MCAO [75, 76]. Brain tissue with viable mitochondrial was stained dark red while the infarcted brain region remained white. After 24 h of reperfusion following MCAO, animals were euthanized by isoflurane anesthesia followed by cervical dislocation. The brain was quickly extracted and sectioned into 1 mm thick slices using McIlwain Tissue Chopper. Brain slices were then incubated in a 2% solution of TTC in phosphate-buffered saline (PBS) for 5 min at 37 °C. Images of brain slices were scanned as previously described [75] and analyzed for infarct and swelling using image analysis software (Image J1.50i, National Institutes of Health, Bethesda, Md, downloadable from (http://rsb.info.nih.gov/ij/download.html). We measured three areas of each brain slice: infarct area (X) (mm^2^), area of the infracted (ipsilateral) hemisphere (Y) (mm [2]), and area of the noninfarcted (contralateral) hemisphere slices (Z) (mm^2^). The % infarct area in the ipsilateral brain hemisphere and brain swelling ratio for the brain sections were calculated by the following equations: (X/Y) *100 and (Y–Z)/Z, and later averaged for each brain.

#### Enzyme-linked immunosorbent assay

Blood samples collected from JUUL/TS/control mice with or without MCAO were analyzed by Quantikine ELISA kits (R and D systems, Minneapolis, MN, USA) for the quantitative determination of thrombomodulin and IL-6 according to the procedure per the manufacturer's protocol.

#### Western blot

JUUL/TS/control mice brain without MCAO (normoxia) or contralateral and ipsilateral brain hemispheres 24 h after MCAO were lysed using RIPA buffer to isolate protein lysate. Protein concentrations of isolated protein lysates were determined using bicinchoninic acid (BCA) assay. Exactly 30 μg of protein from each sample was loaded and separated using a 10% Tris–glycine polyacrylamide precast gel (Bio-Rad Laboratories, Hercules, CA; Cat# 4568034). This method has been used previously to analyze Western blot immunoreactivity [20, 78]. Protein samples were then transferred to a polyvinylidene difluoride membrane (Thermo Fisher; Cat# IPVH00010), and then membranes were incubated in blocking buffer (1% Tween-20 containing Tris-buffered saline (TBST) with 5% bovine serum albumin) to block the nonspecific protein bands for 2 h at room temperature. Membranes were incubated with rabbit polyclonal anti-ZO-1 antibody (1: 2000, Thermo Fisher; Cat# 40-2200), rabbit polyclonal anti-claudin-5 antibody (1: 2000, Thermo Fisher; Cat# 34-1600), rabbit polyclonal anti-occludin antibody (1: 1000, Thermo Fisher; Cat# 40-4700), rabbit polyclonal anti-MMP-9 (N-terminal) antibody (1:1000, Proteintech; Cat# 10375-2-AP), mouse monoclonal anti-ICAM-1 antibody (1: 500, Thermo Fisher; Cat# MA5407), rabbit polyclonal anti-Nrf2 antibody (1: 2000, Thermo Fisher; Cat# PA5-88084), and mouse monoclonal anti-beta-actin antibody (1: 10000 MilliporeSigma; Cat# A5441) in TBST with 5% bovine serum albumin at 4 °C overnight. After 4 times washing with TBST for 15 min each, membranes were incubated with anti-rabbit (Sigma Aldrich; Cat# GENA934- 1ML, RRID: AB_2722659) or anti-mouse (Sigma Aldrich; Cat# GENXA931-1ML, RRID: AB_772209) IgG-horseradish peroxidase secondary antibody (1:10000) in TBST with 5% bovine serum albumin for 2 h at room temperature. After 4 times of 15 min wash with TBST, the protein signals were detected by enhanced chemiluminescence-detecting reagents (Thermo Fisher; Cat# 34577) and visualized in X-ray films in the dark. The protein bands were quantified relative to beta-actin in Image J software.

#### Immunofluorescence

Immunofluorescence staining was performed as previously described with modifications [79]. Mice were euthanized by isoflurane overdose 24 h after MCAO. The brains were sectioned at 30 µM of thickness, fixed with 4% paraformaldehyde (Thermo Fisher) for 15 min, then permeabilized with 0.1% Triton X-100 for 10 min. After washing with the phosphate-buffered saline (PBS), the sections were blocked for 1 h and incubated overnight with primary antibodies for ZO-1 (1:100, Thermo Fisher), Nrf-2 (1:100, Thermo Fisher), claudin-5 (1:100, Thermo Fisher), occludin (1:100, Cell Signaling Technology). Fluorescent secondary antibodies (Thermo Fisher) were used at 1:200 dilutions for 1 h. 4ʹ,6-Diamidino-2-Phenylindole, Dihydrochloride (DAPI) was included for nuclear staining. The images (20X magnitude) were captured with a Nikon A1R multi-photon confocal microscope (Nikon Instrument). Mean total fluorescence intensity was calculated for each color channel using NIS elements AR software, and the intensity of the green color (ZO-1/Nrf2/occludin) or red color (claudin-5) was expressed relative to the blue color (DAPI). Three microscopic fields out of each ipsilateral and contralateral section were used to evaluate the expression levels of ZO-1 and Nrf2.

## Statistical analysis

The sample size for the animal study was estimated based on our previously published literature [12, 15]. No sample size calculation was performed, and there were no sample size differences between the beginning and end of the experiments. No test for normality was performed. All data are expressed as the mean ± S.D. except for Fig. 2 and Fig. 3A, C, which are presented as box and whisker plots. The values were analyzed by one-way analysis of variance with Tukey’s post hoc multiple comparisons (Prism, version 7.0; GraphPad Software Inc., San Diego, CA). P values less than 0.05 were considered statistically significant.

## Supplementary Information

**Additional file 1: Fig. S1.** Full-length western blot images developed by X-ray films. The figures depict full length images of the cropped western blot images used in the original manuscript for figure 6A, 6E, 6I, 7A, and 7E representing brain expression of ZO-1, claudin-5, occludin, Nrf2, and ICAM-1, respectively along with beta actin. The portion of the full-length images which were cropped to represent normoxia, contralateral, and ipsilateral brain expression of control, JUUL, and tobacco smoke (TS)-exposed mice after MCAO are marked with a red line.

## Data Availability

All data generated or analyzed during this study are included in this published article (and its Supplementary Information files).

## Abbreviations

ARRIVE: Animal research: reporting of in vivo experiments
BBB: Blood–brain barrier
CCA: Common carotid artery
CORESTA: Cooperation Centre for Scientific Research Relative to Tobacco
DAPI: 4ʹ,6-diamidino-2-phenylindole, dihydrochloride
ECA: External carotid artery
e-Cig: Electronic cigarette
ICA: Internal carotid artery
MMPs: Matrix metalloproteinases
PBS: Phosphate-buffered saline
SCSM: Single cigarette smoking machines
TBI: Traumatic brain injury
TBST: Tween-20 containing Tris-buffered saline
TJ: Tight junction
tMCAO: Transient middle cerebral artery occlusion
TS: Tobacco smoke
TTC: 2,3,5-Tripheyltetrazolium chloride
USB: Universal serial bus
VEGF: Vascular endothelial growth factor
