# Supplementary material for: Short-term exposure to JUUL electronic cigarettes can worsen ischemic stroke outcome
Source: Fluids Barriers CNS. 2022 Sep 9;19:74. doi: 10.1186/s12987-022-00371-7 (PMC9463848; doi:10.1186/s12987-022-00371-7)
Supplement: Supplementary file 1 — Additional file 1: Fig. S1. Full-length western blot images developed by X-ray films. The figures depict full length images of the cropped western blot images used in the original manuscript for figure 6A, 6E, 6I, 7A, and 7E representing brain expression of ZO-1, claudin-5, occludin, Nrf2, and ICAM-1, respectively along with beta actin. The portion of the full-length images which were cropped to represent normoxia, contralateral, and ipsilateral brain expression of control, JUUL, and tobacco smoke (TS)-exposed mice after MCAO are marked with a red line. [file 12987_2022_371_MOESM1_ESM.pdf]

Supplementary Figure S1.

Fig 6A.

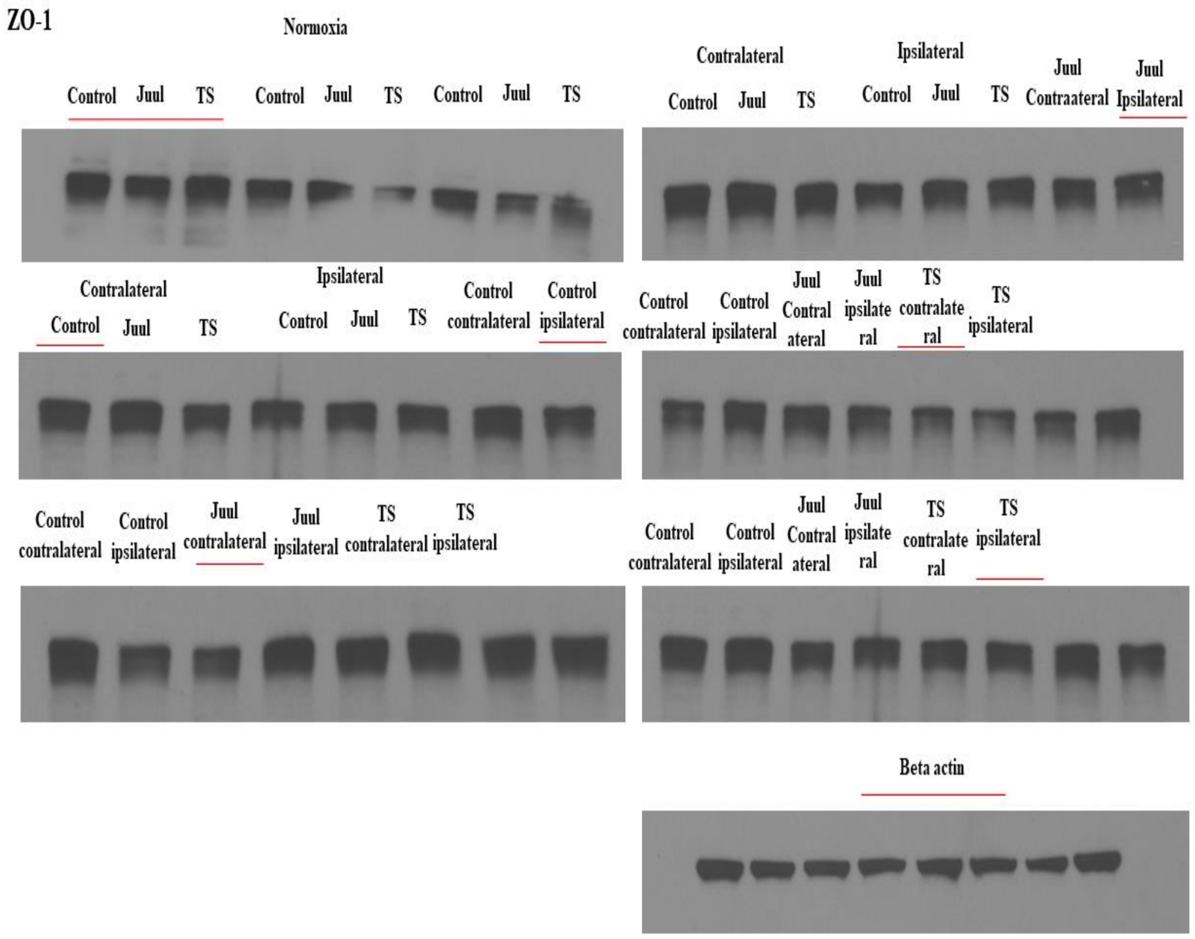

Fig 6E.

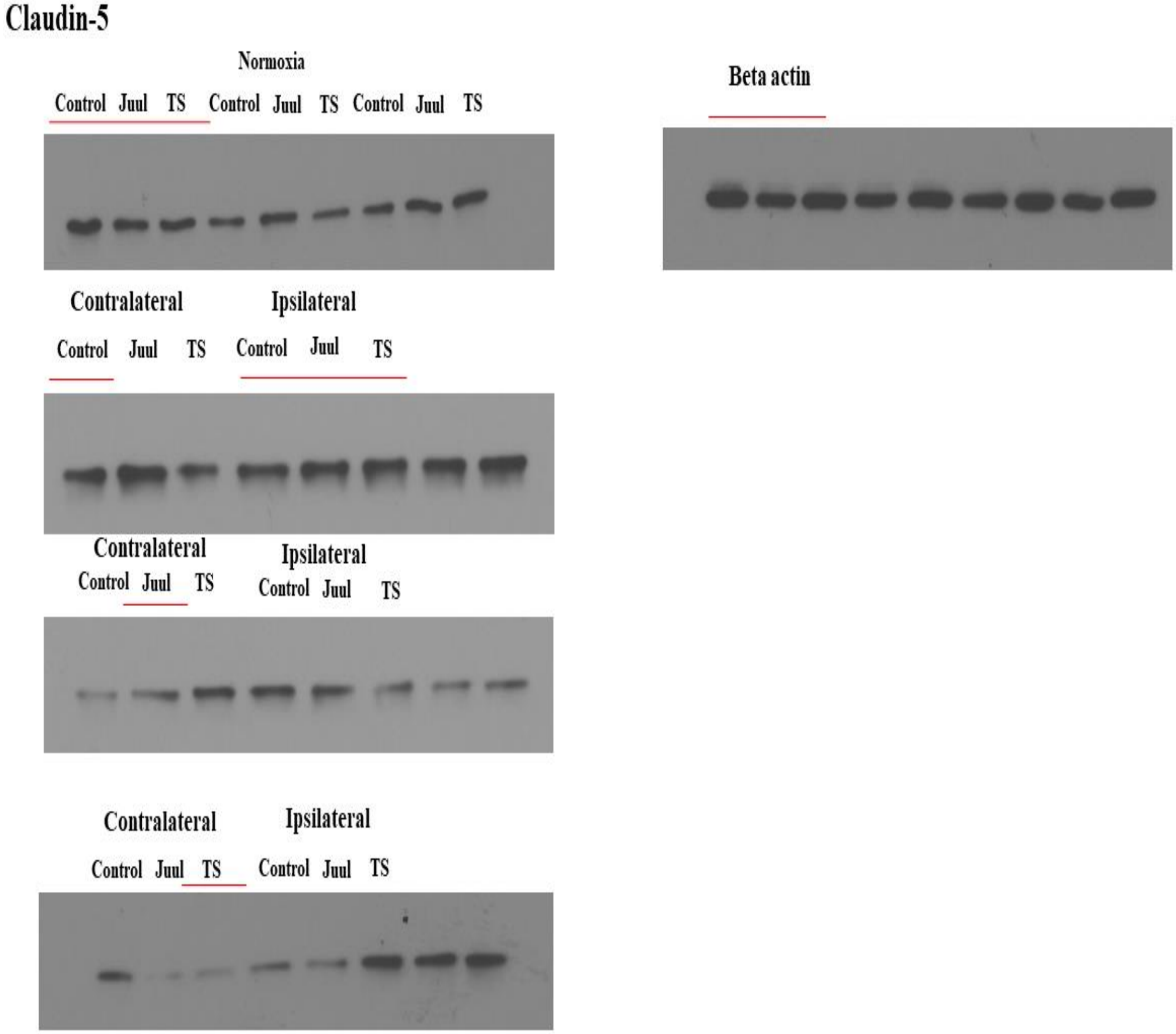
Claudin-5
Normoxia
Control Juul TS Control Juul TS Control Juul TS
Beta actin
Contralateral
Ipsilateral
Control Juul TS Control Juul TS
Contralateral
Ipsilateral
Control Juul TS Control Juul TS
Contralateral
Ipsilateral
Control Juul TS Control Juul TS

Fig 6I.

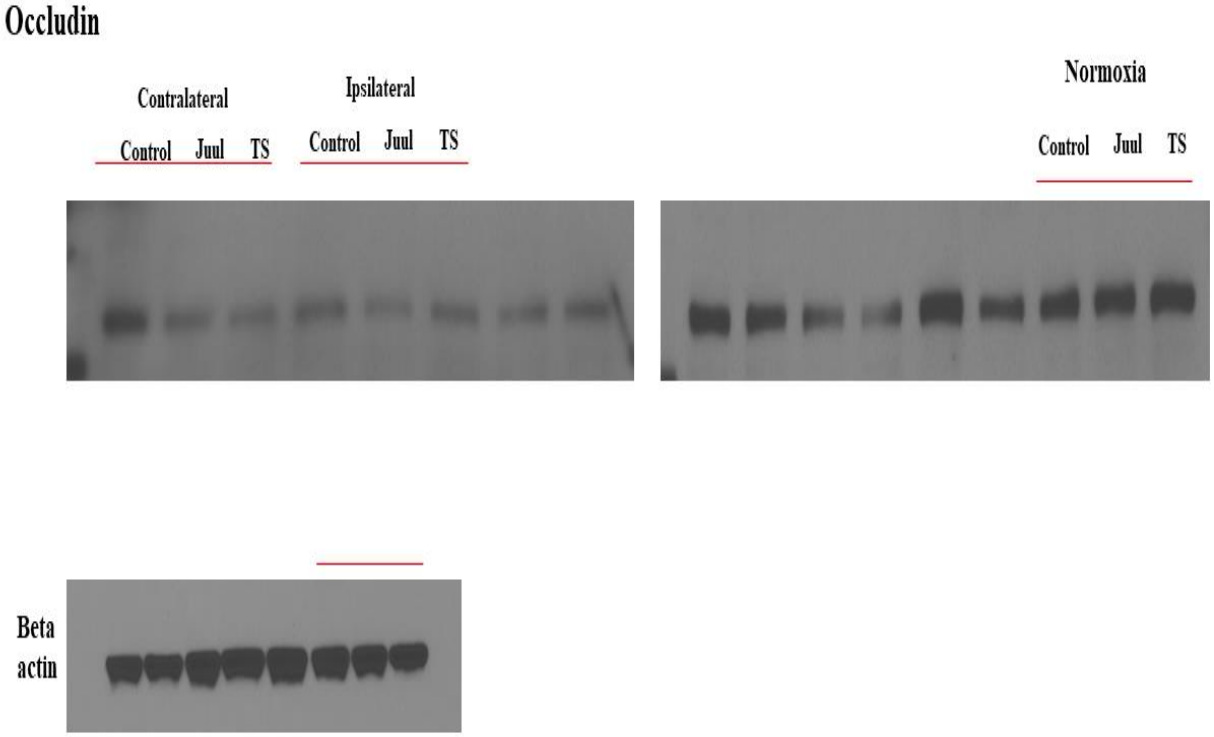
Occludin
Contralateral
Control Juul TS
Ipsilateral
Control Juul TS
Normoxia
Control Juul TS
Beta actin

Fig 7A.

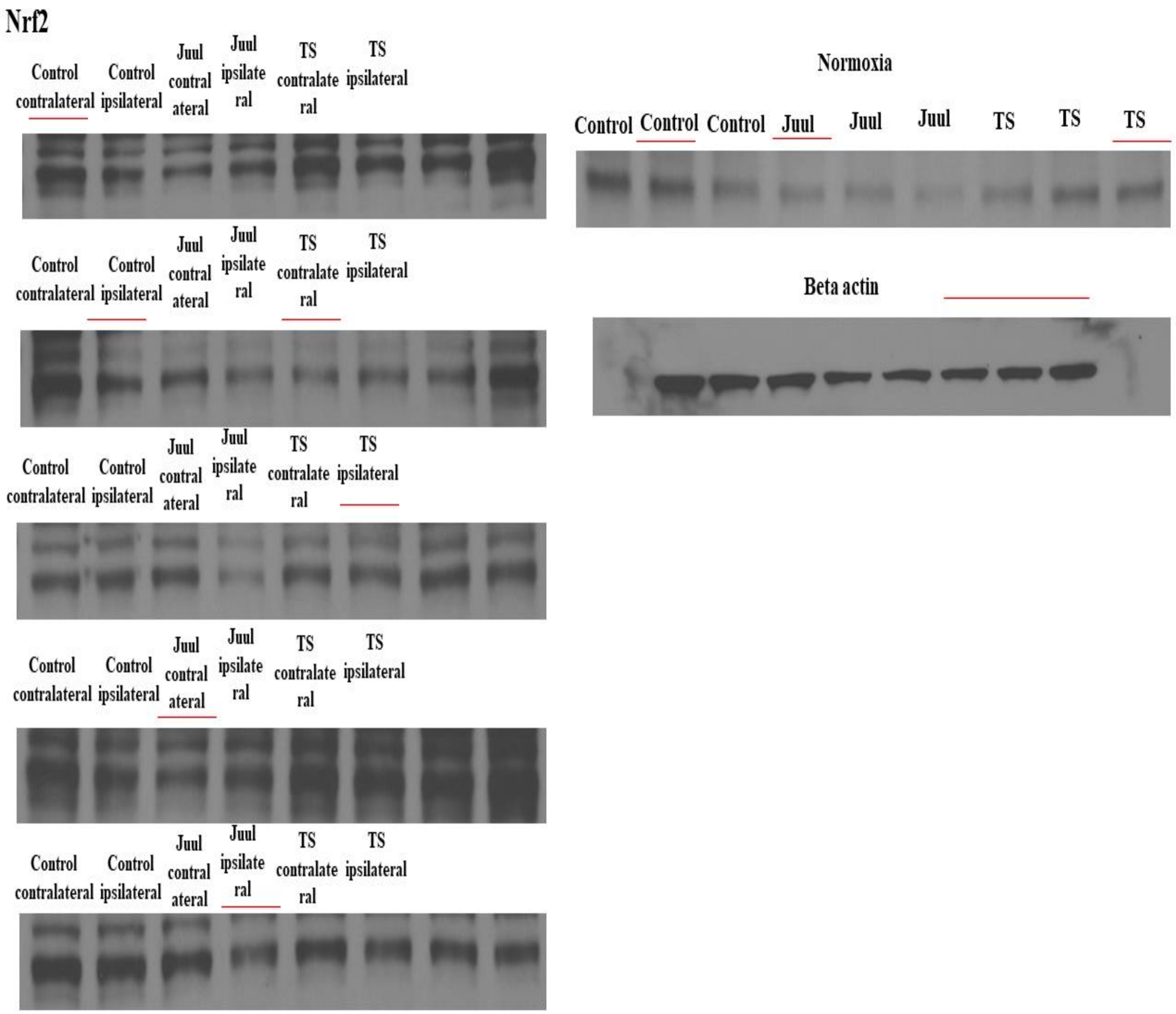
Nrf2
Control contralateral
Control ipsilateral
Juul contral ateral
Juul ipsilate ral
TS contralate ral
TS ipsilateral
Normoxia
Control
Control
Control
Juul
Juul
Juul
TS
TS
TS
Beta actin

Fig 7E.

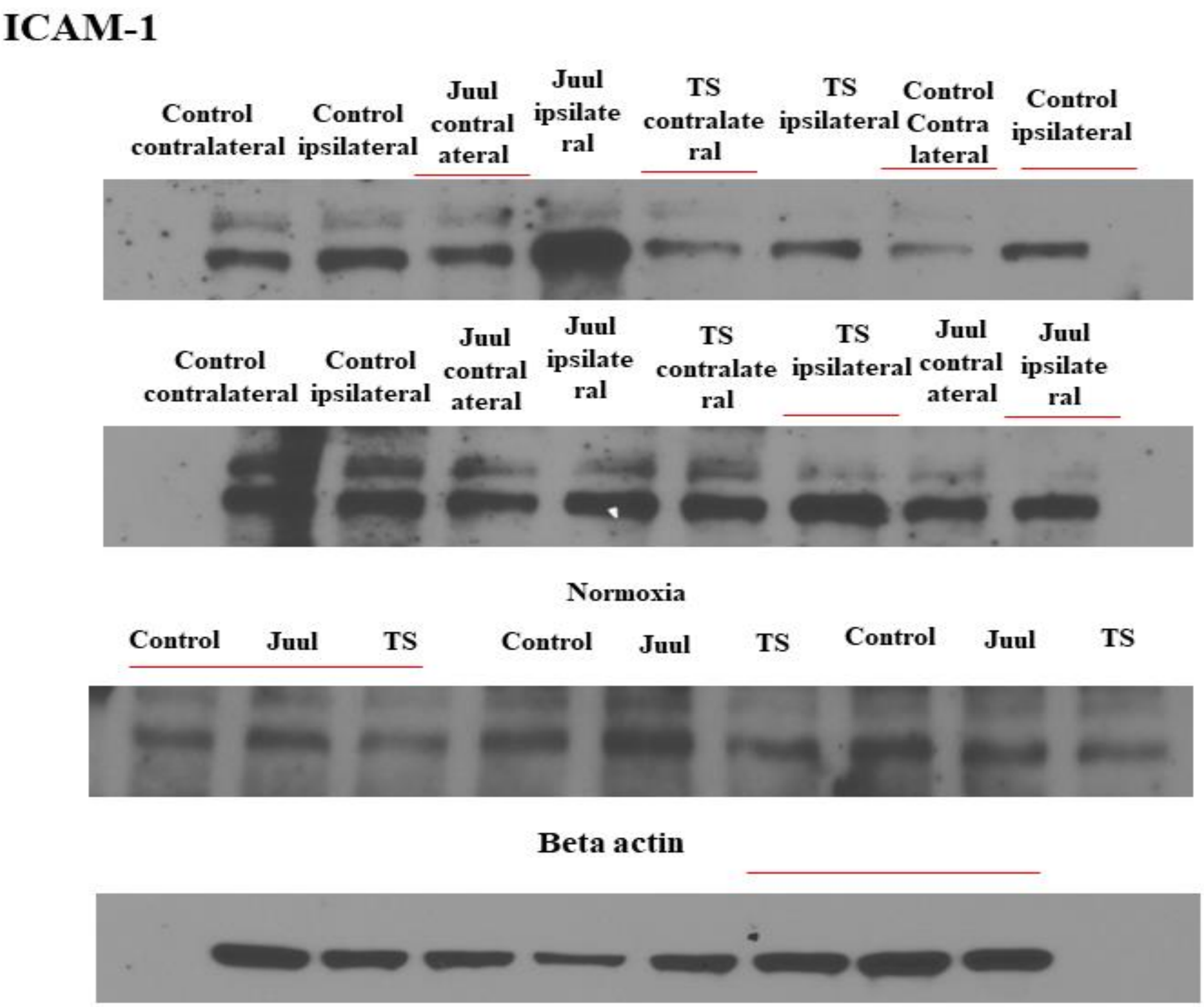
ICAM-1
Control contralateral
Control ipsilateral
Juul contral ateral
Juul ipsilate ral
TS contralate ral
TS ipsilateral
Control Contra lateral
Control ipsilateral
Control contralateral
Control ipsilateral
Juul contral ateral
Juul ipsilate ral
TS contralate ral
TS ipsilateral
Juul contral ateral
Juul ipsilate ral
Normoxia
Control
Juul
TS
Control
Juul
TS
Control
Juul
TS
Beta actin

Supplementary Figure S1. Full-length western blot images developed by X-ray films. The figures depict full length images of the cropped western blot images used in the original manuscript for figure 6A, 6E, 6I, 7A, and 7E representing brain expression of ZO-1, claudin-5, occludin, Nrf2, and ICAM-1, respectively along with beta actin. The portion of the full-length images which were cropped to represent normoxia, contralateral, and ipsilateral brain expression of control, JUUL, and tobacco smoke (TS)-exposed mice after MCAO are marked with a red line.
